# Electrochemical preparation of bismuthene quantum dots for the selective electroreduction of O_2_ to peroxide

**DOI:** 10.1039/d5ra07326j

**Published:** 2025-12-10

**Authors:** Bikash Ranjan Isaac, Sangram Mohapatra, Subbiah Alwarappan, Vijayamohanan K. Pillai

**Affiliations:** a Department of Chemistry, Indian Institute of Science Education and Research Tirupati, Srinivasapuram, Yerpedu Mandal Tirupati Andhra Pradesh 517619 India vijay@iisertirupati.ac.in; b Electrodics and Electrocatalysis Division, CSIR-Central Electrochemical Research Institute Karaikudi Tamilnadu-630003 India

## Abstract

Bismuthene quantum dots (BiQDs), as a zero-dimensional (0D) material, offer significant potential in electrocatalysis due to their quantum confinement, edge effects, and unique electronic structure. Herein, we report a facile electrochemical exfoliation strategy for the synthesis of crystalline BiQDs (lateral dimensions of 2.2 nm) in *N*-methylpyrrolidone (NMP) at room temperature. Their electrocatalytic activity towards the oxygen reduction reaction (ORR) in alkaline media is systematically evaluated. The observed Tafel slope of approximately 40 mV dec^−1^ and the high exchange current density (6.0 × 10^−8^ A cm^−2^) indicate notable kinetic improvements over conventional Pt-based electrocatalysts and enhanced cycling stability. BiQDs exhibit enhanced ORR performance following a two-electron transfer mechanism. This work highlights the promising role of BiQDs as efficient and environmentally friendly ORR electrocatalysts.

## Abstract 2

Bismuthene quantum dots undergo oxygen reduction reaction *via* a selective 2-electron route to produce H_2_O_2_ over the normal 4-electron route that produces water.

## Graphical abstract

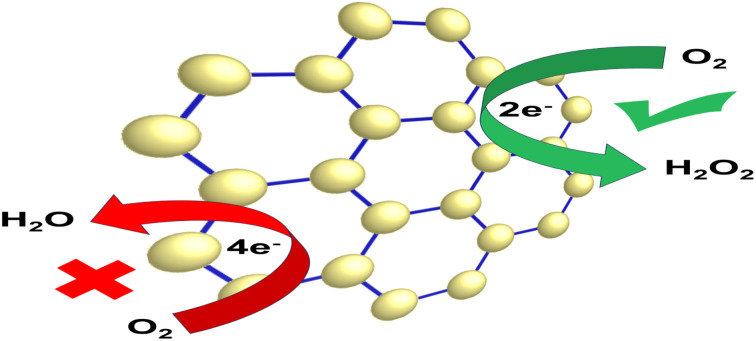

## Introduction

The oxygen reduction reaction (ORR) is a crucial process in energy conversion technologies such as fuel cells and metal-air batteries.^1^ Fuel cells remain a focal point of attention for their eco-friendly energy generation through the electrochemical oxidation of fuels (hydrogen, methanol, or ethanol).^2^ Their efficiency is largely determined by the oxygen reduction reaction (ORR), making the choice of catalyst crucial. Despite the exceptional performance of platinum-based catalysts, their high cost and scarcity limit their widespread applications.^3^ For example, the titanium nitride nanorod arrays as supports for the platinum–palladium–cobalt electrode, tested as a single-cell cathode with a low Pt loading (66.7 µg cm^−2^), demonstrated a higher mass-specific power density (5.85 W mg^−1^) than commercial gas diffusion electrodes (2.46 W mg^−1^) and exhibited superior durability by retaining 72.9% of its initial electrochemically active surface area after 2000 potential cycles, compared with only 59.5% retention by commercial counterparts.^4^ In this context, metal-free or non-noble metal-based catalysts with abundant availability and good catalytic activity are highly desirable.

Two-dimensional (2D) materials have garnered attention for electrocatalysis due to their high surface-to-volume ratio and tunable electronic properties.^5^ Beyond graphene, a wide range of 2D materials, such as transition metal dichalcogenides (*e.g.*, MoS_2_ and WS_2_), black phosphorus (phosphorene), graphitic carbon nitride (g-C_3_N_4_), MXenes, and monoelemental materials, like bismuthene and stanene, have demonstrated remarkable electrocatalytic performance in reactions such as hydrogen evolution, oxygen reduction, and carbon dioxide reduction.^6,7^ These materials exhibit defect chemistries and surface terminations, enabling precise tuning of their catalytic behavior for specific applications. Among these, bismuthene, in which a monolayer of bismuth atoms is arranged in a buckled honeycomb structure, has emerged as a promising candidate for catalysis.^8^ Bismuth (Bi) is non-toxic, relatively inexpensive, features diverse electronic structures, and exhibits intriguing properties such as high electron mobility (1.2 × 10^8^ cm^2^ V^−1^ s^−1^), strong spin–orbit coupling (1.5 eV near the *L* point of the band structure), environmental stability, and a tunable bandgap (0.3–1.0 eV).^8–10^ Bi is also well-known for its underpotential deposition (UPD) behavior, with the Bi/Bi^3+^ couple showing distinct multi-step adsorption and reduction pathways in both aqueous and non-aqueous electrolytes, influenced by factors such as surface structure, coverage, and co-adsorption dynamics. Recent studies, including those in ionic liquids, like [BMIM]BF_4_, reveal that while the overall electrosorption valency remains similar to that in aqueous systems, differences in Bi^3+^ solvation, anion coordination, and co-adsorption processes lead to markedly different UPD mechanisms and coverage transitions.^11^ Emerging theoretical studies have also suggested that bismuth-based nanostructures can serve as efficient ORR catalysts due to their appropriate adsorption energy for oxygenated intermediates. For example, the Bi-110 and Bi-102 surfaces are positioned close to the peak of the activity volcano plot, indicating that bismuth is highly active for the two-electron oxygen reduction reaction (2e^−^ ORR) pathway. Furthermore, bismuth exhibits favorable selectivity toward hydrogen peroxide (H_2_O_2_) production, attributed to its lower Δ*G*(H_2_O_2_) − Δ*G*(O*) values. The Bi-110 surface outperforms other p-block metals and even surpasses state-of-the-art Pt–Hg electrocatalysts in both activity and selectivity for H_2_O_2_ generation.^12^ Building on these findings, incorporating quantum dots (QDs) derived from 2D bismuthene offers a promising strategy to further enhance catalytic performance through quantum confinement effects, increased active sites, and modified electronic structures.^13^ However, most methods for synthesizing BiQDs involve complex chemical processes or high-temperature treatments, which alter the morphology and active site distribution. For example, Son *et al.* used a solvothermal approach, where Bi nanocrystals with tunable sizes (6–27 nm) are synthesized by reducing highly reactive bismuth dodecanethiolate formed from bismuth neodecanoate and dodecanethiol in octadecene using tri-*n*-octylphosphine (TOP) at temperatures as low as 80 °C, with size control achieved by adjusting the aging temperature and time.^14^ Moreover, Verma *et al.* synthesized Bi quantum dots using a laser ablation technique, where a Bi precursor was ablated in water containing sodium lauryl sulfate, with QD formation indicated by a color change.^15^ Therefore, electrochemical route presents a promising approach for synthesizing BiQDs, as it can overcome several of the aforementioned limitations. Since improving the efficiency of reactions in ORR is crucial for advancing fuel cell technology, leveraging such promising materials obtained from electrochemical methods could also enhance catalytic performance and reaction kinetics.

The oxygen reduction reaction (ORR) is a key electrochemical process, but its slow rate remains a major obstacle to the widespread adoption of fuel cell technology. This multielectron reaction involves multiple elementary steps and intermediate species, which vary based on the final product and whether the oxygen–oxygen (O–O) bond breaks. The prevailing theory suggests that in an alkaline aqueous solution, oxygen can be reduced directly to water through a four-electron transfer without the formation of detectable intermediates:O_2_ + H_2_O + 4e^−^ → 4OH^−^ (*E*^0^ = 0.401 V *vs.* SHE)

Alternatively, the reaction may proceed through a two-step pathway where hydroperoxide ions (HO_2_^−^) are generated as an intermediate, which can diffuse into the bulk solution, producing hydroxyl ions (OH^−^):^16^O_2_ + H_2_O + 2e^−^ → HO_2_^−^ + OH^−^ (*E*^0^ = −0.065 V *vs.* SHE)HO_2_^−^ + H_2_O + 2e^−^ → 3OH^−^ (*E*^0^ = 0.867 V *vs.* SHE)Here, we present a room-temperature electrochemical exfoliation to synthesize BiQDs in *N*-methyl-2-pyrrolidone (NMP). The UV and PL results demonstrate that these QDs show optical properties that vary depending on their excitation. Moreover, the resulting BiQDs were characterized by transmission electron microscopy (TEM) and Fourier-transform infrared (FT-IR) spectroscopy, revealing the morphological characteristics of QDs and ORR activity, revealing performance metrics that position them as promising electrocatalysts.

**Scheme 1:**
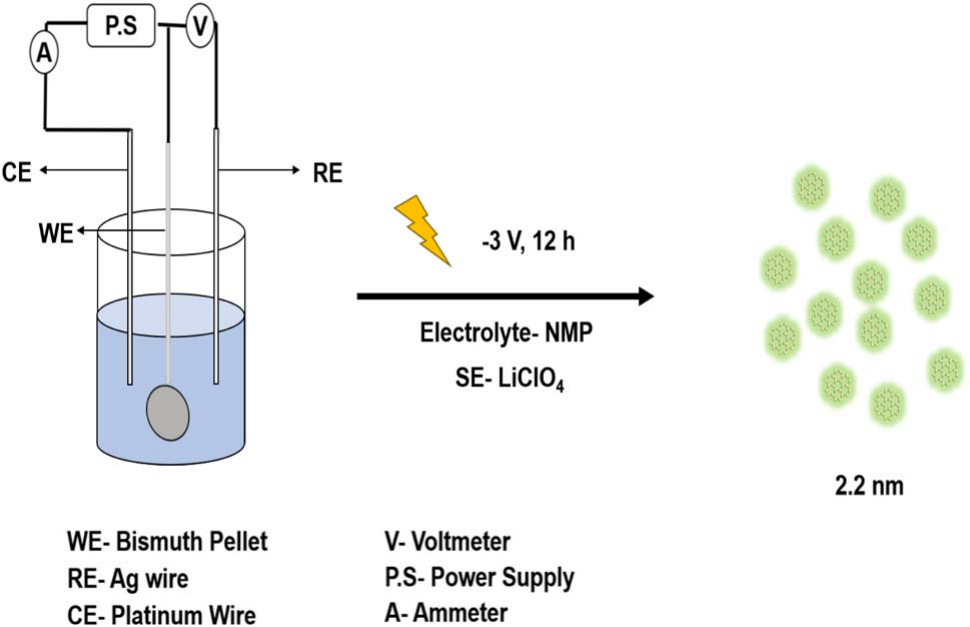
Pictorial representation of the electrosynthesis of BiQDs using 0.1 M LiClO_4_ in NMP and Bi pellet.

Scheme 1 represents the electrosynthesis of BiQDs carried out in a three-electrode cell with a bulk bismuth pellet serving as the working electrode (WE), a silver wire reference (RE), and a platinum wire counter electrode (CE) immersed in *N*-methyl-2-pyrrolidone containing 0.1 M LiClO_4_. Prior to extended reduction, a brief anodic conditioning step at +1.0 V *vs.* Ag wire for 30 min is applied to oxidatively dissolve Bi^0^ into Bi^3+^ (in S1, which shows a clear oxidation wave onset near +0.311 V and an open-circuit potential of −0.46 V). The applied anodic potential, significantly exceeding the standard Bi^3+^/Bi^0^ redox potential (0.308 V), ensures kinetically favorable and sustained generation of Bi^3+^ ions, thereby establishing a well-defined precursor state for subsequent nucleation events. This corresponds to the standard redox potential of Bi^3+^/Bi and facilitates the formation of soluble or surface-confined bismuth oxide species. Also, a thin layer of bismuth species is coated over the Pt foil, which indicates Bi^3+^ formation. A small cathodic peak observed at −0.710 V corresponds to the reduction of Bi^3+^ to metallic Bi^0^, confirming the presence of bismuth oxide intermediates. A pronounced reduction peak at −2.32 V is attributed to the electrochemical exfoliation process, driven by strong cathodic polarization, which facilitates the formation of quantum-confined BiQDs. Thereafter, a constant cathodic potential of −3.0 V was maintained for 12 h to drive the nucleation and controlled growth of ultrafine BiQDs directly from the *in situ* generated Bi–O species. This two-step protocol, *i.e.*, initial anodic Bi dissolution, followed by deep cathodic reduction, ensures a steady supply of bismuth oxide and rapid quantum-dot formation, yielding particles with an average diameter of 2.2 nm. NMP, an organic carbonate electrolyte, is well-suited for lithium-based salts and is commonly used in battery applications.^17^ To prevent oxygen dissolution despite using a non-aqueous electrolyte, argon purging was maintained throughout the experiment.

## Results and discussion

**Fig. 1:**
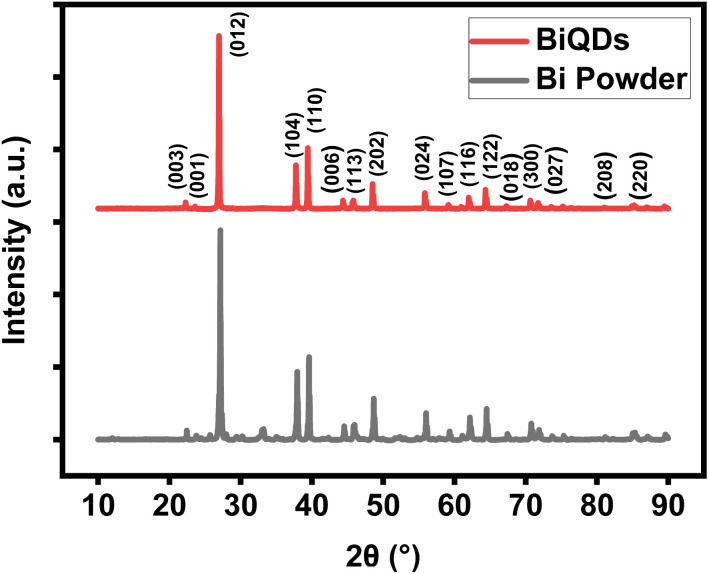
Comparison of the powder XRD patterns of BiQDs and bismuth powder.

Fig. 1 shows powder XRD patterns of BiQDs (red) and bulk Bi (black), with sharp diffraction peaks corresponding to the peaks observed at (012), (104), and (110) planes, which are consistent with the rhombohedral phase of bismuth (JCPDS Card No. 5-519) [space group: *R*3 *m* (166)]. This is in agreement with earlier reports on Bi nanostructures.^18^ The broadening and slight shifts in peaks indicate reduced crystallite size and strain effects, typical of quantum dots.^19^ Notably, the preferential exposure of the (012) and (104) planes has been associated with enhanced electrochemical activity in Bi-based catalysts.^20^

**Fig. 2:**
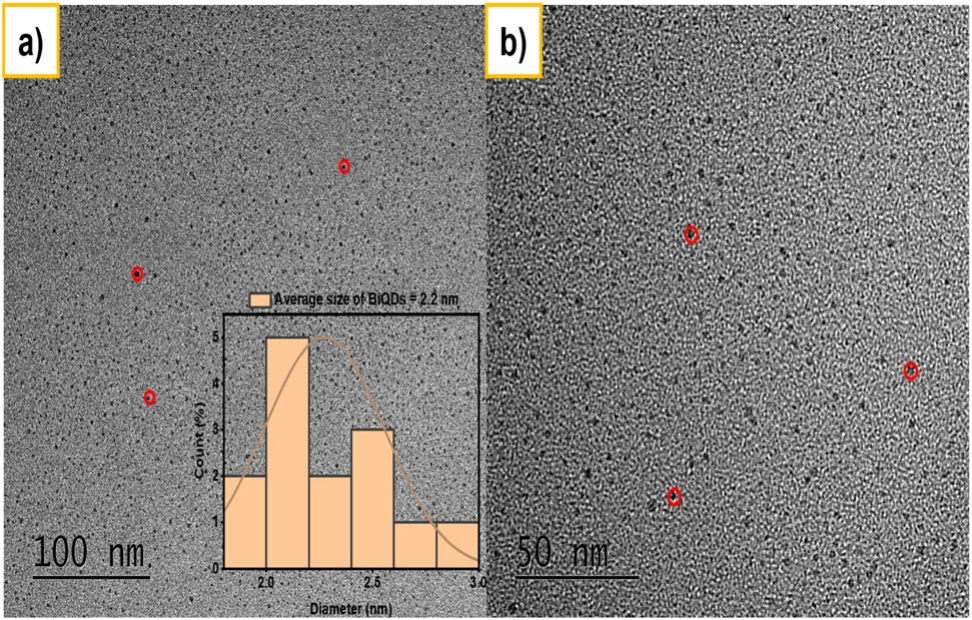
Transmission electron micrographs of BiQDs with an average size of 2.2 nm; (a) inset indicates the distribution while (b) indicates image with a higher resolution.

Transmission electron microscopy (TEM) is employed to investigate the morphology and size distribution of BiQDs. Representative low- and high-magnification TEM images are presented in Fig. 2. The micrographs reveal a high density of uniformly dispersed BiQDs without significant aggregation, indicating excellent colloidal stability. The average particle diameter is determined to be approximately 2.2 nm, consistent across different regions of the sample. The high-resolution image (Fig. 2b) further confirms the nanoscale dimensions and homogeneity of BiQDs. Red circles highlight the selected particles for clarity. The absence of larger particulates or clusters suggests a successful synthesis of discrete, ultra-small quantum dots.

**Fig. 3:**
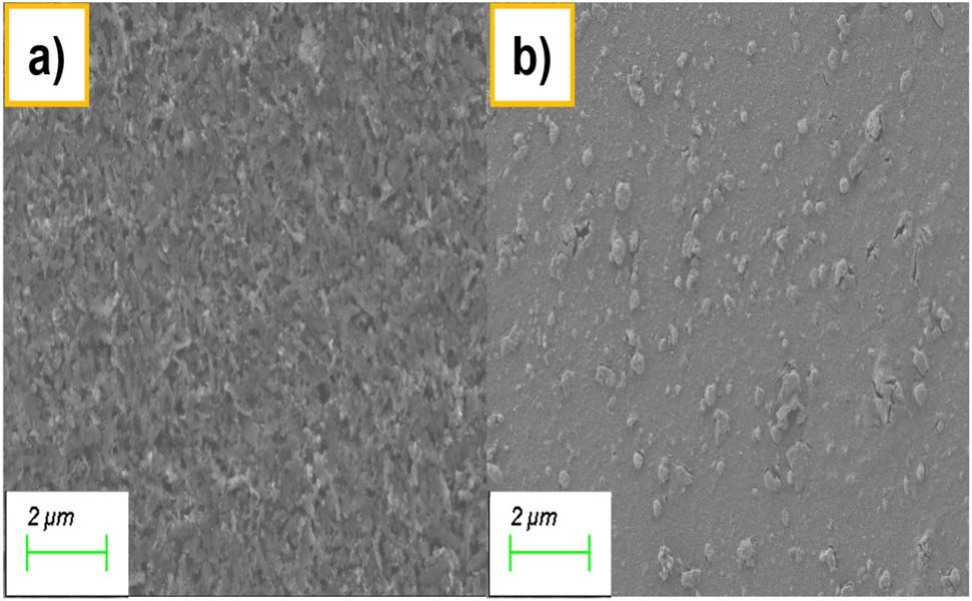
Scanning electron micrographs of (a) BiNS after applying 1 V for 30 min and (b) BiQDs formed on BiNS after applying −3 V for 12 h.

Fig. 3 shows SEM images illustrating the morphology of bismuth during the electrochemical process. As shown in Fig. 3a, the surface is covered with dense, interconnected bismuthene nanosheets (BiNS) obtained after applying 1 V for 30 minutes, which provide a rough and highly textured morphology. This is in agreement with bismuthene nanosheets synthesized electrochemically by applying −1 V in 1 M KHCO_3_ as the electrolyte.^21^ In contrast, Fig. 3b reveals the transformation of these nanosheets into discrete BiQDs upon applying a potential of −3 V for 12 hours. The nanosheets are decorated with uniformly distributed spherical nanostructures, confirming the successful conversion of BiNS into BiQDs. This morphological transition highlights the potential of controlled electrochemical production of bismuth nanostructures for advanced applications.

**Fig. 4:**
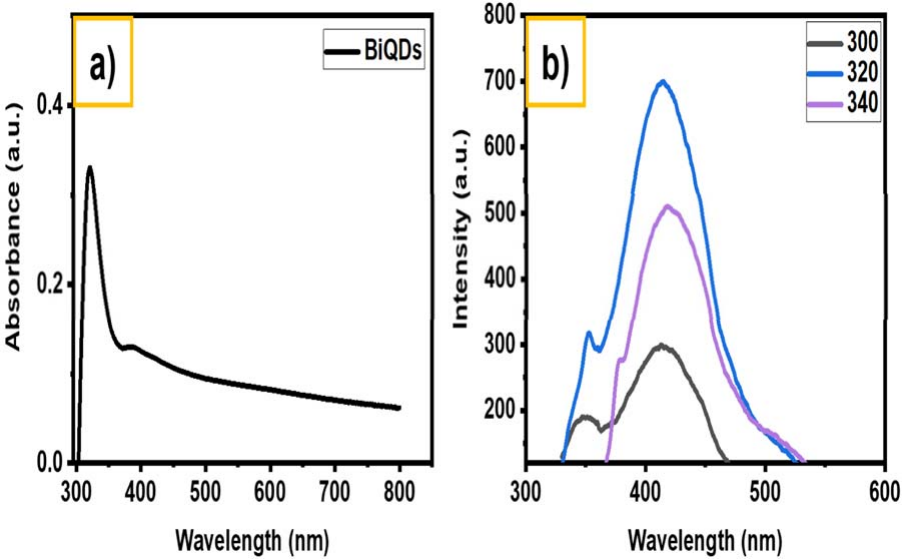
(a) UV-vis absorption spectra and (b) photoluminescence spectra of green BiQDs.

The optical properties of the green-emitting BiQDs are characterized using UV-vis absorption and photoluminescence (PL) spectroscopy, as shown in Fig. 4. The UV-vis absorption spectrum (Fig. 4a) exhibits a strong absorption band centered around 320 nm, indicative of quantum confinement effects arising from the small size of BiQDs. The absorbance steadily decreases with increasing wavelength, consistent with the typical semiconductor quantum dot behavior. The PL spectra (Fig. 4b) demonstrate excitation-dependent emission, with the maximum photoluminescence intensity observed upon excitation at 320 nm, yielding an emission peak centered around 430 nm, suggesting their potential applicability in optoelectronic and bioimaging applications.^22^ Compared to BiQDs synthesized by solvothermal methods, which exhibit broadband absorption up to 850 nm and are optimized for self-powered UV photodetection, the electrochemically synthesized green-emitting BiQDs show a sharper absorption band at 320 nm and strong excitation-dependent photoluminescence centered at 430 nm, indicative of stronger quantum confinement.^23^

**Fig. 5:**
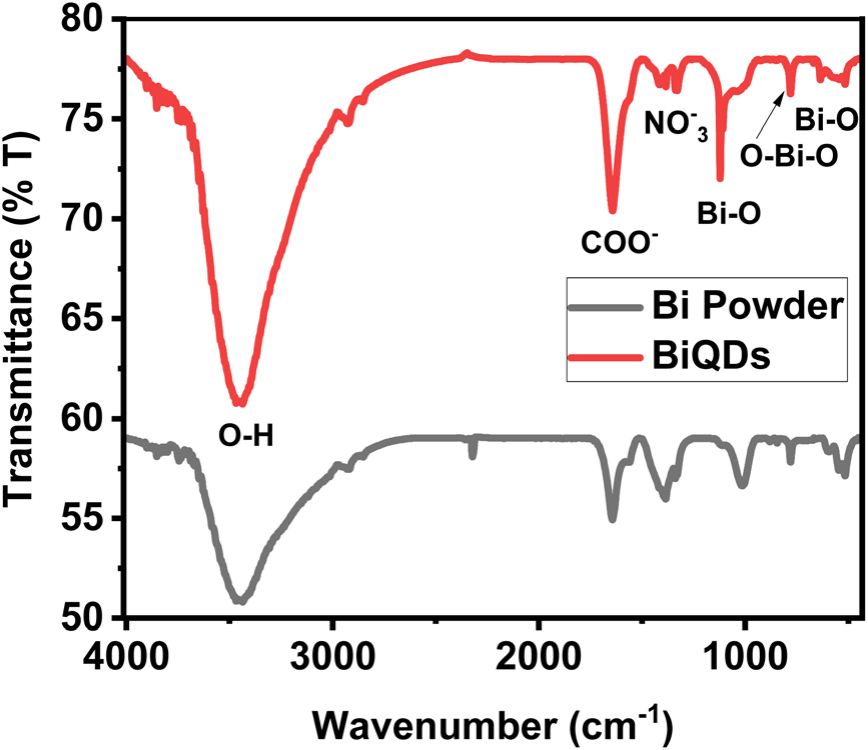
FT-IR spectra of BiQDs and bismuth powder.

Fourier-transform infrared (FT-IR) spectroscopy is used to investigate the surface functional groups and chemical composition of BiQDs in comparison with that of bulk Bi powder, as shown in Fig. 5. The BiQDs spectrum exhibits a broad absorption band around 3400 cm^−1^, attributed to the O–H stretching vibration of surface-adsorbed hydroxyl groups or water molecules.^24^ Peaks observed at ∼1570 cm^−1^ and ∼1380 cm^−1^ correspond to asymmetric and symmetric stretching vibrations of carboxylate (COO^−^) groups, indicating the presence of surface-bound oxidation products formed from the electrochemical degradation of the NMP solvent under the applied potential. Additionally, the peak at ∼1350 cm^−1^ is assigned to NO_3_^−^ vibrations, suggesting nitrate residues from the precursor. The weak nitrate peak is likely attributable to surface contamination, possibly arising from environmental adsorption during electrosynthesis. Distinct peaks in the region of 800–500 cm^−1^ correspond to Bi–O and O–Bi–O stretching modes, confirming the formation of bismuth-oxide bonds in the quantum dot structure.^25^ In contrast, the Bi powder spectrum shows significantly less defined features, indicating the absence of these surface functionalities. These results support the successful surface modification and formation of Bi–O-rich BiQDs during the synthesis process. These functional groups enhance the colloidal stability of nanodots in polar media and facilitate better interfacial charge transfer during electrochemical reactions. Moreover, they introduce additional surface-active sites, which can significantly improve catalytic performance.

**Fig. 6:**
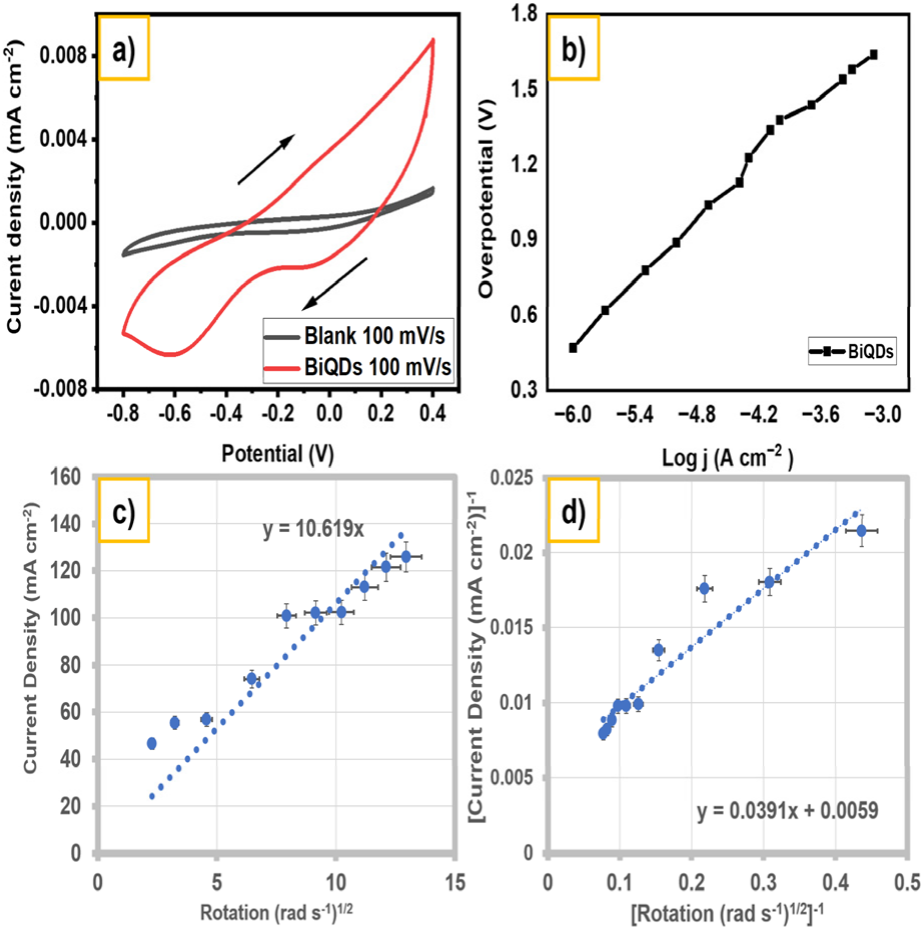
(a) Comparison of cyclic voltammograms in O_2_-saturated 1.0 M KOH (pH 13.92) at 100 mV s^−1^ in the potential window of −0.8 V to 0.4 V; working electrode: glassy carbon, counter electrode Pt wire, and reference electrode: Hg/HgO, and the third cycle is shown for all g-CNQDs. A blank (baseline) was taken with argon-purged KOH. (b) Tafel plot, (c) Levich plot, and (d) Koutecky–Levich plot for BiQDs in 1.0 M KOH.

The electrocatalytic ORR activity of BiQDs is assessed in O_2_-saturated 1.0 M KOH (pH 13.9). As shown in Fig. 6a, the third-cycle cyclic voltammogram of BiQDs (red trace) exhibits a pronounced cathodic wave with an onset potential near −0.15 V *vs.* Hg/HgO and a peak current density of −7.5 mA cm^−2^ at −0.8 V, whereas the argon-purged blank (gray trace) shows negligible current, confirming the ORR origin of the redox feature. The corresponding overpotential *vs.* log *j* plot (Fig. 6b) yields a Tafel slope of 40 mV dec^−1^, indicative of a rate-determining first electron transfer step with an exchange current density of. 6.0 × 10^−8^ A cm^−2^. RDE measurements at rotation rates from 50 to 1600 rpm (Fig. 6c) produce a linear relationship between current density (*j*) and *ω*^1/2^ (slope = 10.61), demonstrating diffusion-limited kinetics. The calculated diffusion coefficient is approximately 7.45 × 10^−5^ cm^2^ s^−1^. Koutecky–Levich analysis (Fig. 6d) further affords a linear *j*^−1^*vs. ω*^−½^ plot with a slope of 0.039, corresponding to an apparent two-electron transfer pathway for ORR. Bismuth-based electrocatalysts favor the 2-electron ORR pathway due to their low energy barrier for the *OOH intermediate formation, as revealed by density functional theory (DFT) calculations.^26^ This facilitates selective peroxide production, while the 4-electron pathway remains energetically less favorable due to poor O–O bond cleavage and weak adsorption of oxygenated intermediates.^12^ Moreover, the electrochemically exfoliated BiQDs exhibit a low Tafel slope of 40 mV dec^−1^ and an exchange current density of 6.0 × 10^−8^ A cm^−2^, demonstrating superior charge-transfer kinetics compared to many benchmark non-noble metal catalysts. In particular, carbon-based quantum dots, NiAl-layered double hydroxides, and doped NiO systems typically achieve similar 2e^−^ selectivity only through complex doping or coordination-engineering strategies.^27–29^ In contrast, the present BiQDs attain comparable or enhanced activity through intrinsic quantum confinement and surface oxygen-rich functionalities generated under ambient electrochemical conditions. The ambient, scalable synthesis route and the unique electronic structure of BiQDs collectively enable high selectivity toward peroxide formation, underscoring their promise as a sustainable alternative for 2e^−^ ORR catalysis. Taken together, these results establish BiQDs as efficient and selective catalysts for the two-electron reduction of oxygen in alkaline media.

## Conclusions

In summary, we have demonstrated a two-step protocol at room temperature for the electrochemical exfoliation synthesis of BiQDs (2.2 nm) in *N*-methyl-2-pyrrolidone/0.1 M LiClO_4_. Comprehensive characterization (TEM, UV-vis, PL, FT-IR) confirms the formation of crystalline, oxide-rich QDs with strong quantum confinement and surface-bound functional groups. When deployed as ORR electrocatalysts in alkaline media, BiQDs exhibit a low onset potential, Tafel slope of 40 mV dec^−1^, and Koutecky–Levich analysis reveals a selective two-electron pathway. These results position BiQDs as cost-effective, earth-abundant alternatives for selective peroxide generation and underscore the broader potential of zero-dimensional p-block materials in energy conversion applications.

## Experimental section

### Electrochemical synthesis

A bismuth pellet weighing 1.11 g was prepared from bismuth powder (SRL, Bismuth Metal Powder extrapure, 99%, −325 mesh). Electrosynthesis was carried out to obtain BiQDs by applying a constant potential of +1 V for 30 minutes and −3 V for 12 hours. The setup included a platinum wire as the counter electrode and a silver wire serving as the quasi-reference electrode, with 0.1 M LiClO_4_ dissolved in NMP as the electrolyte. Following the synthesis, the samples were centrifuged in order to separate them from the electrolyte. A yield of 8% ± 3% BiQDs was obtained. The optical properties were studied using UV-visible and photoluminescence (PL) spectroscopy at room temperature. Structural features and size were characterized through Fourier-transform infrared (FT-IR) spectroscopy, powder X-ray diffraction (PXRD), and X-ray photoelectron spectroscopy (XPS). Particle sizes were further confirmed by transmission electron microscopy (TEM).

### Electrocatalysis

Electrochemical measurements were performed using a three-electrode configuration. The working electrode consisted of a glassy carbon electrode (3 mm diameter) coated with 40 µL of a quantum dot suspension (1 mg mL^−1^), while a platinum wire acted as the counter electrode and a pre-calibrated Hg/HgO electrode served as the reference. Experiments were carried out in 1.0 M KOH saturated with oxygen, with control (blank) measurements conducted in 1.0 M KOH purged with argon. Cyclic voltammetry was performed within a potential window of −0.8 V to 0.4 V at varying scan rates. Galvanostatic steady-state polarization studies were conducted in oxygen-saturated 1.0 M KOH to evaluate exchange current densities and apparent Tafel slopes. Koutecky–Levich analysis was carried out using linear sweep voltammetry at different electrode rotation speeds, employing a platinum rotating disk electrode (RDE) coated with the quantum dot suspension, a platinum coil as the counter electrode, and a pre-calibrated Hg/HgO reference electrode, all in 1.0 M KOH electrolyte.

## Conflicts of interest

There is no conflict to declare.

## Supplementary Material

RA-015-D5RA07326J-s001

## Data Availability

The data underlying this work are available in the published article and its supplementary information (SI). Supplementary information is available. See DOI: https://doi.org/10.1039/d5ra07326j.
