# Supplementary material for: Electrochemical preparation of bismuthene quantum dots for the selective electroreduction of O2 to peroxide
Source: RSC Adv. 2025 Dec 10;15(57):49088–93. doi: 10.1039/d5ra07326j (PMC12693423; doi:10.1039/d5ra07326j)
Supplement: RA-015-D5RA07326J-s001 [file RA-015-D5RA07326J-s001.pdf]

Supplementary Information (SI) for RSC Advances.
This journal is © The Royal Society of Chemistry 2025

Electrochemical Preparation of Bismuthene Quantum Dots for Selective Electroreduction of O_2 to Peroxide

*Bikash Ranjan Isaac [a], Sangram Mohapatra [a], Subbiah Alwarappan [b], and Vijayamohanan K. Pillai [a] **

[a] Bikash Ranjan Isaac, Sangram Mohapatra and Vijayamohanan K. Pillai

Department of Chemistry, Indian Institute of Science Education and Research, Tirupati Srinivasapuram, Yerpedu Mandal Tirupati Dist, Andhra Pradesh, India – 517619.

Email: vijay@iisertirupati.ac.in

[b] Subbiah Alwarappan

Electrodics and Electrocatalysis Division, CSIR-Central Electrochemical Research Institute, Karaikudi, Tamilnadu – 630003, India

Email: alwarappan@cecri.res.in

Number of pages: 8

Number of figures: 2

Number of tables: 3

Cyclic Voltammogram

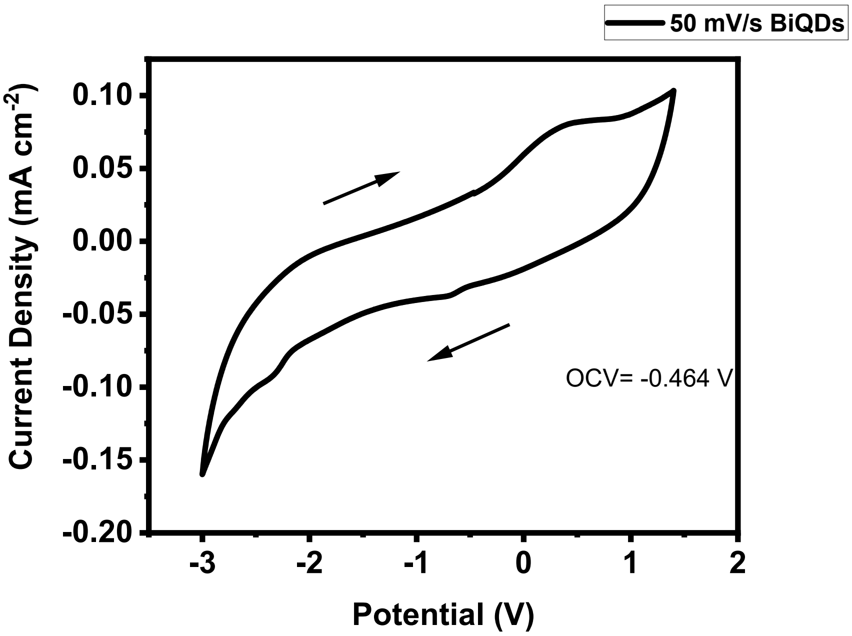

Figure S1. Cyclic Voltammogram of Bi Powder at 100 mV/s scan rate. Electrodes – Working: Bi Pellet, Counter: Platinum Foil, Quasi-Reference: Silver Wire

Figure S1 represents the electrosynthesis of BiQDs carried out in a three-electrode cell with a bulk bismuth pellet serving as the working electrode (WE), a silver wire reference (RE), and a platinum wire counter electrode (CE) immersed in N-methyl-2-pyrrolidone containing 0.1 M $LiClO_4$. Prior to extended reduction, a brief anodic conditioning step at +1.0 V vs. Ag wire for 30 min is applied to oxidatively dissolve Bi^0 into Bi^{3+} (as informed by the cyclic voltammogram in S1, which shows a clear oxidation wave onset near +0.311 V and an open-circuit potential of –0.46 V). This corresponds to the standard redox potential of Bi^{3+}/Bi (0.308 V) for bismuth-based catalysts. Also, a thin layer of bismuth species is coated over the Pt foil., which indicates Bi^{3+} formation. A small cathodic peak observed at –0.710 V corresponds to the reduction of Bi^{3+} to metallic Bi^0, confirming the presence of bismuth oxide intermediates. A pronounced

reduction peak at –2.32 V is attributed to the electrochemical exfoliation process, driven by strong cathodic polarization, which facilitates the formation of quantum-confined BiQDs.

Stability Test:

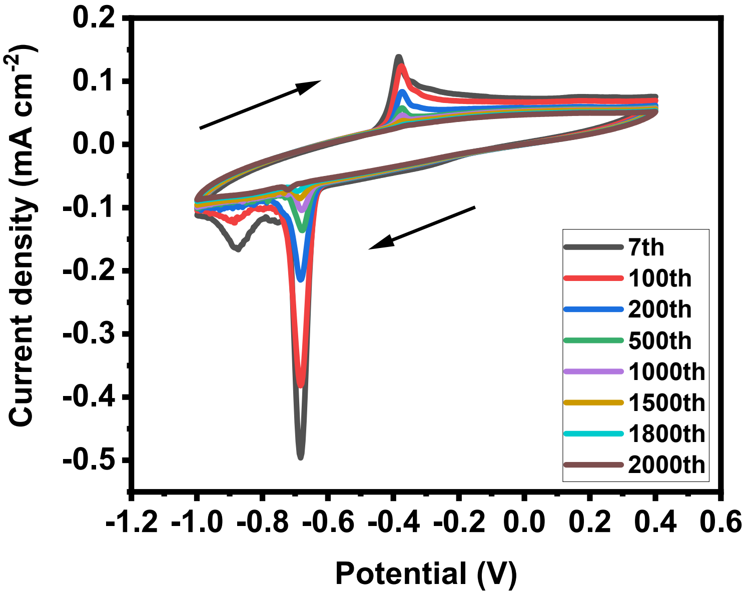

Figure S2. Cyclic Voltammogram of BiQDs at 100 mV/s scan rate for ORR. Electrodes – Working: BiQDs coated on glassy carbon, Counter: Platinum Foil, Reference: Hg/HgO.

Figure S2 shows the cyclic voltammograms (CVs) of BiQDs recorded for 2000 consecutive cycles at a scan rate of 100 mV s^{-1} to evaluate their electrochemical stability toward the oxygen reduction reaction (ORR). The BiQDs-modified glassy carbon electrode exhibits a well-defined cathodic reduction peak near –0.68 V (vs Hg/HgO), corresponding to the reduction of molecular oxygen, and distinct anodic peaks on the reverse sweep. The second, more intense peak at –0.8 V arises from the further reduction of these intermediates to hydroperoxide (HO_2^-) in alkaline medium via a two-electron transfer. These oxidation peaks, appearing between –0.38 V is attributed to the re-oxidation of oxygenated intermediates such as Bi–O, Bi–OH, or Bi_2O_3 species formed during the cathodic reduction process. Their consistent presence and minimal variation in intensity over successive cycles suggest reversible redox transitions

between Bi^0 and Bi^{3+} states, highlighting the participation of surface-bound oxygen species in the ORR mechanism. With increasing cycle number, the CV curves nearly overlap, indicating excellent electrochemical durability and structural stability of the BiQD catalyst during repeated potential cycling. The negligible shift in peak potential and retention of current density even after 2000 cycles confirm that the BiQDs maintain their catalytic activity and resist degradation under continuous operation, demonstrating their suitability for long-term ORR applications.

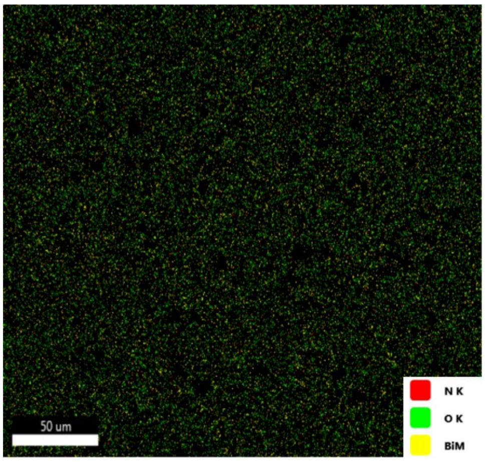

Figure S3. Elemental mapping of BiQDs.

Elemental distribution mapping of BiQDs confirms uniform dispersion of nitrogen, oxygen, and bismuth throughout the sample during electrochemical processing, further supporting their potential application in electrocatalytic systems.

Methods

Electrochemical Kinetic Analyses

Tafel Analysis:

Tafel analysis was performed to determine the kinetic parameters of the electrochemical reaction. The Tafel plots were obtained by plotting the overpotential (η) against the logarithm of current density (log j) in the kinetically controlled region. The slope of the linear region of

the plot provides the Tafel slope (b), which is related to the charge-transfer kinetics and mechanistic pathway of the reaction as seen in Figure 6 b). The Tafel equation is expressed as:

$$\eta = \pm A\, log_{10}(\frac{i}{i_0})$$

where η = overpotential, (V), ± = plus sign denotes an anodic reaction while a minus sign shows cathodic reaction, A = Tafel slope, (V), i = current density, (A/m^2), i_0 = exchange current density, (A/m^2)

Levich Analysis:

The Levich analysis was conducted to evaluate the diffusion-limited current and to estimate the number of electrons transferred (n) during the electrochemical process. The measurements were carried out using a rotating disk electrode (RDE) at varying rotation rates (ω). The Levich equation describes the relationship between the limiting current density (j_L) and rotation rate:

$$i_L = 0.62nFAD^{2/3}\omega^{1/2}v^{1/6}C$$

where F is the Faraday constant (96485 C mol^{-1}), C_0 is the bulk concentration of the electroactive species, D is the diffusion coefficient, ν is the kinematic viscosity of the electrolyte, and ω is the angular rotation rate (rad s^{-1}). A linear i_L vs $\omega^{1/2}$ relationship confirms diffusion-controlled behavior as seen in Figure 6 c).

Koutecký–Levich (K–L) Analysis:

To separate kinetic and diffusion contributions, Koutecký–Levich (K–L) plots were constructed according to:

$$\frac{1}{j} = \frac{1}{j_K} + \frac{1}{B\omega^{1/2}}$$

Where j is the measured current density, j_K is the kinetic current density, and B is the Levich constant defined as:

$$B = 0.62nFAD^{2/3}v^{1/6}C$$

K–L plots of 1/j vs $\omega^{-1/2}$ were used to determine the number of electrons (n) and to verify first-order kinetics with respect to the electroactive species. The kinetic current density (j_K) was obtained from the intercept of the plot as seen in Figure 6 d). The number of electrons calculated was 2.9.

All electrochemical data were collected using a three-electrode setup with a working electrode (platinum RDE), a reference electrode (Hg/HgO), and a counter electrode (Pt wire) in O_2-saturated 1 M KOH. The temperature was maintained at room temperature (∼298 K).

Table S1. Various reported synthetic methods for similar materials:

SN.	Method	Precursor	Absorption Wavelength (in nm)	Size Distribution (in nm) [average]	Ref
1	Hydrothermal Method	$Bi(NO3)_3 \cdot 5H2O$	840 nm	6 nm	1
2	Solvothermal approach	Bismuth neodecanoate	490 nm	26nm	2
3	Solvothermal method	Bismuth neodecanoate	350nm	22 nm	3
4	Alcoholysis route in organic media.	Bi_2O_3 powder	462 nm	3.4–5.1 nm	4
5	Liquid-Phase Exfoliation	Bi powder	264 nm	4.9 ± 1.0 nm	5

6	Laser ablation	99.99% pure metallic bismuth plate	266 nm	6 nm	[6]

Table S2. Comparison of traditional benchmark platinum catalysts with Bismuthene Quantum dots.

**Electrocatalyst**	**KOH**	**Temprature (in 0 °C)**	**Tafel Slope (mV dec^{-1})**	**Exchange Curent density (A cm^{-2})**	**References**
Pt/C	6.0 M	65	44	5 x 10^{-11}	[7]
Rough sputtered Pt	6.0 M	25	50	5 x 10^{-9}	[7]
Smooth sputtered Pt	1.0 M	26.4	45	1 x 10^{-11}	[7]
Bulk Pt	1.0M	26.2	43	3 x 10^{-12}	[7]
Bismuthene Nanosheet	0.1M	25	72.43	-	[8]
Bismuthene Quantum dots	1.0M	25	40	6 x 10^{-8}	This work

Table S3. Comparison of the electrochemical performance, synthesis approach, and unique advantages of the present BiQDs-based oxygen reduction catalyst with recently reported 2e⁻ ORR catalysts.

Catalyst / Synthesis Method	Key ORR Performance	Main Advantages	Comparison with Present Work (BiQDs)	Reference
Carbon-based quantum dots synthesized via ultrasonic/sonochemical route	High selectivity (~95%) for 2e^{-} ORR; H_2O_2 yield ~1466.6 mmol g^{-1} over 12 h	Simple, surfactant-free synthesis; scalable; efficient 2e^{-} ORR catalyst	Our BiQDs offer a metal(loid)-based system with distinct electronic structure and oxygen functional groups, providing enhanced selectivity and stability compared to carbon QDs.	[9]

NiAl-LDH catalyst with Al-induced coordination engineering	Selectivity: 94.23% ± 0.78% at 0.4 V vs RHE; stable over 20,000 cycles	High durability; fine-tuned electronic structure for *OOH stabilization	Our BiQDs use quantum confinement and oxide-rich surface chemistry rather than coordination tuning. They provide a simpler, ambient-condition synthesis and excellent selectivity for peroxide formation.	[10]
NiO catalysts modified by cation (Fe, Co, Cu, Zn) doping	Enhanced activity and 2e⁻ selectivity vs undoped NiO	Electronic structure tuning through controlled doping; stable operation	Our BiQDs similarly exploit surface oxygen functionalities for optimized ORR selectivity. However, BiQDs avoid complex doping processes, achieving comparable performance via electrochemical exfoliation.	[11]
Electrochemical exfoliation of bismuthene to BiQDs in NMP/$LiClO_4$ at room temperature	Onset potential: −0.15 V vs Hg/HgO; Tafel slope: 40 mV dec^{-1}; $2e^-$ pathway confirmed; stable operation (Supplementary Fig. Sx)	Ambient synthesis; scalable; high selectivity and stability; earth-abundant and non-toxic material	Compared to recent reports, BiQDs combine structural simplicity with high activity and selectivity, representing a distinct contribution among $2e^-$ ORR catalysts.	This Work

References:

1 S. S. M. Hassan, M. E. Mahmoud, R. M. Tharwat and A. M. Abdelfattah, *BMC Chem.*, 2024, **18**, 202.

2 H. Pan, H. Chu, Y. Li, Z. Pan, J. Zhao, S. Zhao, W. Huang and D. Li, *J. Mater.*, 2023, **9**, 183–190.

3 J. Zhu, H. Chen, Y. Zi, M. Wang and W. Huang, *Nanotechnology*, 2022, **34**, 025202.

4 H. Zhang, P. Wu, Y. Li, L. Liao, Z. Fang and X. Zhong, *ChemCatChem*, 2010, **2**, 1115–1121.

5 C. Xing, W. Huang, Z. Xie, J. Zhao, D. Ma, T. Fan, W. Liang, Y. Ge, B. Dong, J. Li and H. Zhang, *ACS Photonics*, 2018, **5**, 621–629.
6 R. K. Verma, K. Kumar and S. B. Rai, *J. Colloid Interface Sci.*, 2013, **390**, 11–16.
7 M. F. Weber, M. J. Dignam, S.-M. Park and R. D. Venter, *J. Electrochem. Soc.*, 1986, **133**, 734–738.
8 D. Deng, S. Wu, H. Li, H. Li and L. Xu, *Small*, 2023, **19**, e2205469.
9 Y. Wang, Z. Yang, C. Zhang, Y. Feng, H. Shao, J. Chen, J. Hu and L. Zhang, *Ultrason. Sonochem.*, 2023, **99**, 106582.
10 Y. Zhang, D. Chen, S. Yu, Y. Feng, C. Zhang and J. Hu, *Adv. Funct. Mater.*, DOI:10.1002/adfm.202513626.
11 H. Feng, Y. Song, Y. Zhang, Q. Qi, C. Zhang, Y. Feng and J. Hu, *Chem. Eng. J.*, 2025, **506**, 160364.
